# The Impact of Feedback Modalities and the Influence of Cognitive Load on Interpersonal Communication in Nonclinical Settings: Experimental Study Design

**DOI:** 10.2196/49675

**Published:** 2023-10-05

**Authors:** Chryselle Rego, Enid Montague

**Affiliations:** 1 Jarvis College of Computing and Digital Media DePaul University Chicago, IL United States; 2 Department of Mechanical & Industrial Engineering University of Toronto Toronto, ON Canada

**Keywords:** physician-patient interaction, cognitive load, visual feedback, haptic feedback, postsession feedback

## Abstract

**Background:**

The escalating demands of modern health care systems, combined with the emotional toll of patient care, have led to an alarming increase in physician burnout rates. This burnout, characterized by emotional exhaustion, depersonalization, and reduced personal accomplishment, can hinder doctors’ ability to connect with patients effectively. Moreover, the cognitive load arising from information overload and the need for multitasking can further hinder doctors’ ability to connect with patients effectively. Understanding the complex relationship between physician burnout and cognitive load is crucial for devising targeted interventions that enhance physician well-being and promote effective physician-patient interactions. Implementing strategies to alleviate burnout and cognitive load can lead to improved health care experiences and patient outcomes.

**Objective:**

Our study explores the interplay between physician burnout and its potential impact on interpersonal communication, particularly focusing on the role of cognitive load using a pilot study in a nonclinical setting involving nonclinical participants.

**Methods:**

This study uses an experimental design to evaluate 3 feedback tools (haptic, visual, and postvisit summary) and measure the cognitive load they impose on nonclinical participants in a nonclinical environment. The NASA Task Load Index, a widely accepted measure of cognitive load, was used to quantify the cognitive load associated with the feedback tools. The study used a within-subject design, meaning participants experienced all 3 feedback methods. A sample of 18 nonclinical participants was selected using counterbalancing techniques.

**Results:**

Postsession feedback not only enhancing performance but also mitigating the influence of cognitive load as compared with real-time feedback (haptic+visual). Participants with interview experience showed lower cognitive load levels when exposed to real-time feedback as compared with novice users. In contrast, postsession feedback was more effective for novice users. In addition, cognitive workload emerged as a moderating factor in the relationship between feedback tools and their impact on performance, particularly in terms of speaking balance and pace. This moderating effect suggests that the correlation between feedback tool efficacy and performance varies based on an individual’s cognitive load while using the feedback tool. The comparison of postfeedback with haptic feedback yielded a Z score of −3.245 and a *P* value of .001, while the comparison with visual feedback resulted in a Z score of −2.940 and a *P* value of .003. These outcomes underscore a significant disparity in the means between postsession feedback and real-time feedback (haptic+visual), with postsession feedback indicating the lowest mean score.

**Conclusions:**

Through the examination of various feedback tools, this study yields significant and insightful comparisons regarding their usability and appropriateness in nonclinical settings. To enhance the applicability of these findings to clinical environments, further research encompassing diverse participant cohorts and clinical scenarios is warranted.

## Introduction

### Overview

Effective communication lies at the heart of positive physician-patient interactions, playing a crucial role in achieving improved health outcomes. Poor communication has been linked to detrimental effects on patient well-being, highlighting the significance of addressing this issue in health care [1]. A study conducted by the University of Kansas School of Medicine revealed that patients’ reports of their understanding of the postdischarge information and instructions they received were significantly lower than what their doctors perceived, underscoring the need for enhanced communication strategies [1].

A factor impacting physician-patient interactions is the rising rate of physician burnout [2]. The demands of modern health care systems, coupled with the emotional toll of patient care, have led to an alarming prevalence of burnout among physicians, affecting 54% of them [2,3]. Overall, 66% of physicians have high levels of emotional exhaustion, 33% encounter increased levels of depersonalization, and 39% experience decreased personal accomplishment [4]. As physicians experience emotional exhaustion, depersonalization, and a reduced sense of personal accomplishment, their ability to effectively engage with patients may be compromised. Furthermore, cognitive load arising from information overload and the need for multitasking can further impede physicians’ capacity to process and respond to patient cues, leading to diminished empathetic communication [4]. Understanding the intricate relationship between physician burnout and cognitive load is pivotal in developing targeted interventions to improve physician well-being and foster meaningful and effective physician-patient interactions. Implementing strategies to alleviate burnout and mitigate cognitive load can pave the way for improved health care experiences and patient outcomes [5]. In this context, feedback is a valuable tool for enhancing physician-patient interactions [6].

At present, physicians receive summative feedback in the form of patient-reported experience measures (PREMs). PREMs are a type of health care assessment tool used to collect information about patients’ experiences with the health care services they receive [7]. Unlike patient-reported outcome measures, which focus on the health outcomes and symptoms experienced by patients, PREMs specifically capture patients’ perspectives on the quality of care, communication, interactions with health care providers, and the overall health care environment [8].

PREMs are typically collected through surveys or questionnaires completed by patients after receiving health care services [8]. These surveys ask patients about various aspects of their experience, such as the ease of scheduling appointments, clarity of information provided, attitude of health care professionals, waiting times, and overall satisfaction with the care received. However, the implementation of PREMs in regular care visits and decisions presents major challenges owing to their time-consuming nature, varying patient interpretations, and the complexity of data collection and analysis [9]. Different patients may have varying expectations and interpretations of their experiences, which makes it challenging to obtain standardized and objective measurements. Because PREMs do not provide real-time feedback, they are often not well understood by clinicians, leading to confusion about how to best use PREMs to improve patient care. Patients from different cultural backgrounds and language proficiency levels may interpret questions differently or find it difficult to accurately express their experiences. As a result, concerns about the validity and reliability of surveys, difficulties surrounding interpretation, issues of context, and anxiety surrounding negative feedback have resulted in doctors’ skepticism toward patient surveys as a quality enhancement tool. On exploring the ambiguities in doctors’ attitudes toward patient experience surveys, it was discovered that most physicians undermine the potential for survey-based quality improvement; however, they still find value in receiving patient feedback [9]. This raises the question of whether real-time feedback might serve as a better quality enhancement tool to replace summative feedback received through PREMs.

In light of these pressing issues, this research aims to investigate the interplay between physician burnout and its potential impact on interpersonal communication, particularly focusing on the role of cognitive load, using a pilot study in a nonclinical setting involving nonclinical participants. The pilot study focused on testing the usability and effectiveness of 3 feedback tools (haptic feedback, visual feedback, and postvisit summary) designed to mitigate physician burnout and enhance communication skills. Simultaneously, we measured the cognitive load associated with each feedback modality to assess its potential impact on the effectiveness of communication.

Our methodological adaptations were motivated by several factors. The development and evaluation of a feedback system within clinical settings can be resource intensive in terms of time and expense. Therefore, we attempted to test these feedback modalities in nonclinical settings as a more feasible approach before implementing them in clinical environments. The primary rationale for using nonclinical participants in our experimental study design was the remote nature of the study, which facilitated recruitment from a broader pool. Although our preference was to include primary care physicians as participants, their limited availability poses challenges in recruiting this specific population. Consequently, conducting testing in nonclinical settings allows us to mitigate costs related to uncovering potential flaws by engaging users willing to invest time and effort in finding imperfections in the feedback modalities tested.

In conclusion, this research holds immense promise in addressing the pressing concerns of physician-patient communication and clinician burnout. By identifying effective feedback tools and understanding their impact on cognitive load and communication, our study aims to enhance physician-patient interactions and foster a supportive environment. Through targeted interventions, we envision improved health care experiences and better patient outcomes, ultimately benefiting both the patients and health care providers.

### Research Background

This study is part of an extensive research study that uses human-centered design methodologies to develop and assess the effectiveness of feedback modalities aimed at enhancing physician-patient communication in primary care settings [6]. The primary goal of this system is to facilitate improved interactions between physicians and patients without imposing additional cognitive load on physicians.

Prior investigations have explored conventional feedback approaches and novel feedback methods for physicians [6]. However, only a limited number of studies have evaluated the effectiveness of these feedback tools compared with the cognitive load caused by these modalities.

According to a scenario-based design session conducted in a previous study [6], several factors have been identified as crucial considerations when devising a feedback system: (1) it should not distract physicians during patient interactions, (2) the feedback provided should be easily understandable and implementable, (3) real-time feedback is deemed more effective, (4) the feedback should not add to the cognitive load or contribute to burnout among physicians, and (5) it should foster a balanced conversation between physicians and patients by reducing interruptions and instances where physicians talk more than the patient [10]. On the basis of these essential considerations, three distinct concepts emerged from the scenario-based design process: (1) haptic or tactile feedback, (2) visual feedback using visual cues, and (3) postvisit feedback in the form of a written summary.

## Methods

### Study Design

**Figure 1:**
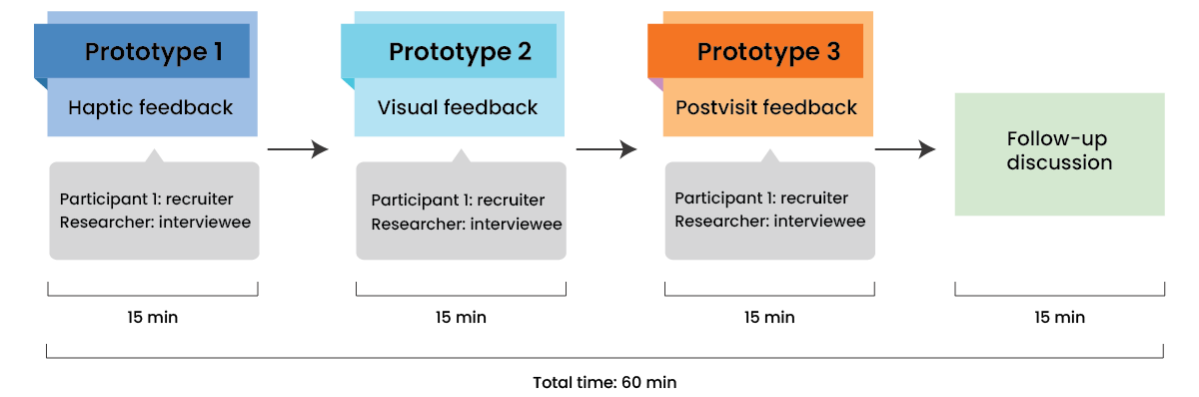
Study design indicating the participant flow through the study activities.

A within-subjects design methodology was used to evaluate the feedback tools used in this study. The session lasted for 60 minutes and was conducted via Zoom (Zoom Video Communications Inc). A concise outline of the study design is shown in Figure 1.

During usability testing, participants assumed the role of an interviewer, whereas the researcher undertook the persona of an interview candidate. A standardized interview script was provided to all participants to facilitate guided communication; however, participants were encouraged to improvise when deemed appropriate. After the session, the participants were asked follow-up questions. A total of 3 successive rounds of interviews were conducted wherein each interview round featured the use of a different feedback tool.

As burnout rate is one of the main factors influencing physician-patient interaction, the cognitive load associated with using the feedback tool was measured using the NASA Task Load Index (NASA TLX). The NASA TLX assesses workload on a 7-point scale, categorized into 5 levels: low (0-9), medium (10-29), somewhat high (30-49), high (50-79), and very high (80-100). It uses 6 dimensions to assess mental demands, physical demands, temporal demands, performance, effort, and frustration. Increments in high, medium, and low estimates for each point resulted in 21 gradations on the scales.

The NASA TLX is a cognitive workload assessment tool that allows users to perform subjective workload assessments of individuals working with various systems or interfaces [11]. After testing each feedback tool, participants were assessed using a digital version of the NASA TLX. Notably, none of the participants expressed objections or encountered difficulties while completing the questionnaire.

### Statistics and Data Analysis

In this study, the data analysis process included the application of specific statistical tests to assess the suitability and reliability of the scales used for factor analysis. The Kaiser-Meyer-Olkin (KMO) test and Bartlett sphericity test were used to determine the appropriateness of the scale for factor analysis. The KMO test assesses sampling adequacy by measuring the proportion of variance that can be attributed to underlying factors. A KMO value above 0.6 indicates suitability for factor analysis, whereas a value above 0.8 suggests high suitability. Similarly, the Bartlett sphericity test evaluates the hypothesis that the intercorrelations among the variables are all equal to 0. A significant result from this test indicated that the variables were correlated, supporting the appropriateness of the factor analysis.

In addition, to measure the scale’s reliability, Cronbach α coefficient was calculated, which is widely considered the optimal method for evaluating internal consistency. A Cronbach α score above .8 indicates excellent internal consistency, whereas scores between .5 and .8 imply good consistency. By conducting these statistical tests, we ensured the robustness of our data and reliability of the scale, enhancing the validity and rigor of our study’s findings.

### Hypothesis

Cognitive workload moderates the relationship between the usability of the feedback tools and its impact on physician-patient interaction such that their association will become weaker or stronger, depending on how high or low a physician’s cognitive load rating is while using the feedback tool. This is because cognitive workload plays a critical role in influencing an individual’s capacity to receive, process, and implement feedback effectively. Given that physicians are required to assimilate and apply feedback while simultaneously engaging in patient interactions, a lower cognitive load induced by the feedback tool is positively associated with improved usability and effectiveness of the feedback tool in enhancing physician-patient interaction.

### Ethics Approval

The research protocol adhered to the ethical standards set forth by DePaul University’s institutional review board (research protocol #IRB-2022-547), ensuring compliance with the established guidelines.

### Study Sample Size

Using a counterbalancing approach, a participant cohort comprising 18 individuals (n=18) was selected using the Communication & Digital Media Participant Pool. All conceivable orders were used to prevent biases and control the effects of cognitive load and other variables on the study findings.

All participants underwent a screening process, resulting in the inclusion of 18 students who met the specific criteria and were subsequently invited to participate in the study. Participants from various academic disciplines, including human-centered design, accounting, computer science, and communication programs, were invited to participate. The participant pool was assembled from a diverse population and encompassed individuals with varied backgrounds and experiences. The median age of the participants was 26 (range 19-30) years. Although most participants possessed experience in interviewee roles, only 5 participants had prior experience conducting interviews. All participants, except for 3, were unfamiliar with real-time feedback tools.

### Assigning Participants to Groups

A within-subjects design, alternatively referred to as a repeated-measures design, was used, wherein each participant sequentially evaluated the 3 feedback tools, and their performance with each feedback tool was assessed. To eliminate bias, a completely randomized design was used, wherein each participant was selected at random to participate in the usability test.

### Study Activities

#### Haptic Feedback

In the initial interview round, the primary objective was to gain comprehensive insights into the interviewees’ background while engaging in discussions about their job roles and responsibilities. To foster interactivity, participants role-playing as interviewers were actively encouraged to inquire about the interviewee’s qualifications, experience, and suitability for their position.

Given the remote nature of the study, haptic cues were systematically generated through the use of the participants’ mobile phones or smartwatches. The settings with touchscreen and smartwatch devices were customized to emit tactile sensations that corresponded to the distinct communication behaviors under examination. The process involved mapping specific communication parameters, such as pauses, active listening moments, increased pace, and the lack of articulation to the corresponding haptic sensations. For instance, a steady but prolonged vibration might signify the need for a pause and was used to indicate the need to practice speaking balance through active listening and pausing to ask the interviewee questions. In contrast, a brief series of rapid pulses can indicate instances of interruption. As a result, it was used to alert participants to slow down their pace and articulate better while interacting with the interviewee.

Previous studies have provided an understanding of human perceptual capabilities in the field of vibrotactile displays [12]. Pasquesi and Gorlewicz [12] delineated 3 specific frequency ranges that produce distinct perceptual effects through vibration. A frequency range below 3 Hz corresponds to a slow kinesthetic motion evoking a gradual pulsation; the 10-70 Hz range creates a fluttering sensation similar to a tapping or rapid pulse; and finally, a 100-300 Hz frequency engenders a seamless vibration similar to a steady buzz [12]. To simplify the learning curve associated with haptic feedback, only 2 haptics were used in this study. A rapid pulse of 10-70 Hz, also known as the “heartbeat” vibration, was used to get the participants to demonstrate improved pace and articulation, while the steady buzz, also known as the “quick” vibration of 100-300 Hz, was used to encourage participants to display improved speaking balance by pausing and asking the interviewee questions.

**Figure 2:**
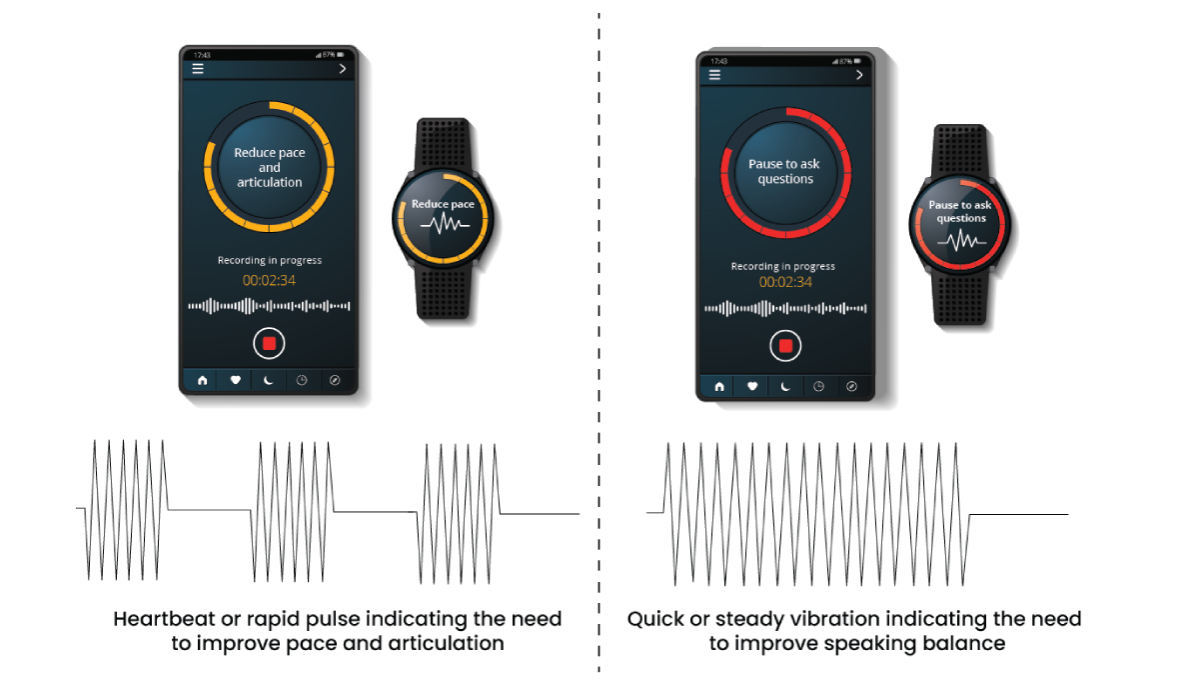
Visual representation of haptic feedback as transmitted via smartphone and smartwatch.

The interpretation of physiological data related to speech metrics is rooted in our aim to comprehensively assess the impact of haptic cues on communication dynamics and cognitive load [13]. By scrutinizing objective speech parameters, such as speech rate, pauses, and speaking balance, we sought to objectively quantify the efficacy of haptic feedback in influencing communication behaviors. A visual representation of the haptic feedback is shown in Figure 2.

#### Visual Feedback

**Figure 3:**
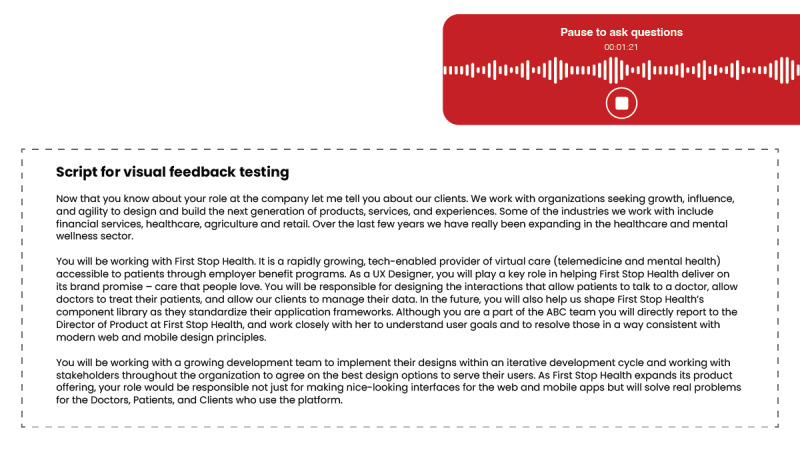
Visual representation of the visual feedback. UX: user experience.

During the second round of the interview phase, participants assumed the role of an interviewer and were tasked with conveying details regarding the company’s background, client base, and service offerings to the interviewees. During this session, participants received real-time dynamic feedback using visual cues. Throughout this interaction, visual cues in the form of color-coded instructions were used to provide feedback on pace, articulation, and speaking balance. Specifically, the use of stoplight colors (red, yellow, and green) was implemented to facilitate easy recognition and recall. Red signified the need to pause and ask questions, yellow indicated the need to reduce pace, and green connoted excellent pace and articulation. A visual representation of the visual feedback is shown in Figure 3.

#### Postsession Feedback

**Figure 4:**
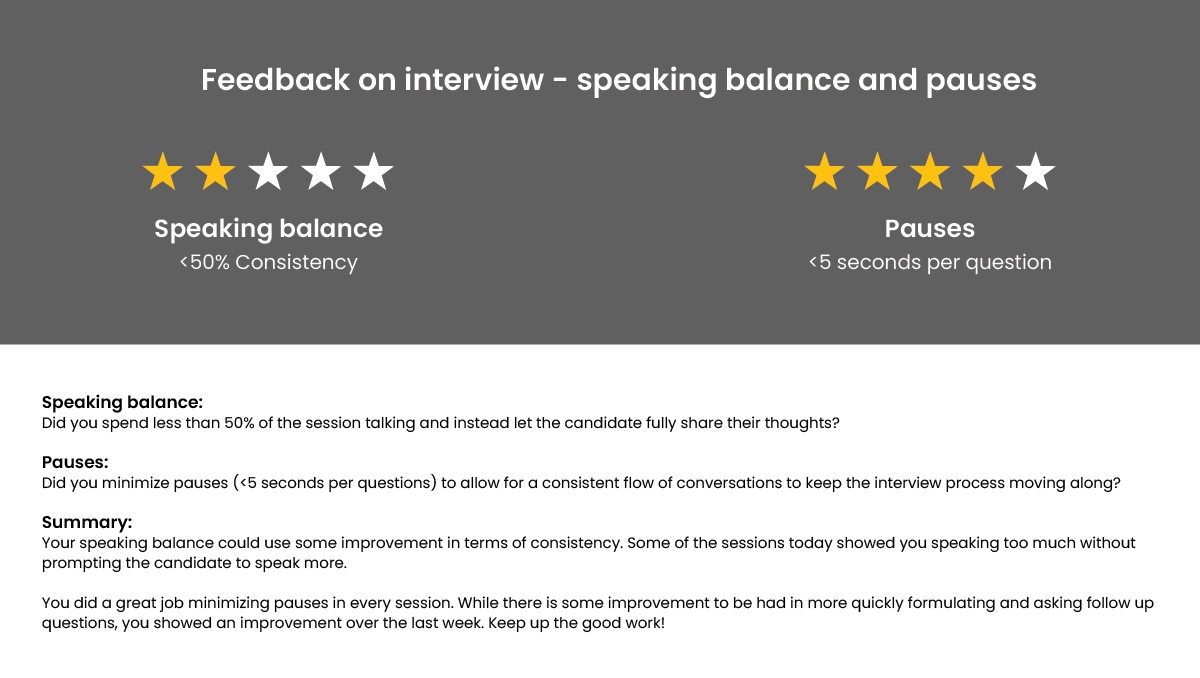
Visual representation of the postsession feedback.

In the concluding segment of the interview, participants role-played as an interviewer and were tasked with briefing the interviewee with final instructions and employment prerequisites. During this interview phase, the participants did not receive any feedback during the conversation. They were expected to improvise based on their previous learning. At the end of the session, the participants were provided with postsession feedback articulated in the format of a written synopsis. This feedback consisted of an overall evaluation of their performance, illustrated through a rating mechanism coupled with a detailed summary outlining their overall proficiency in interpersonal communication, notably encompassing parameters of pace, articulation, and speaking balance. A visual representation of the postsession feedback is shown in Figure 4.

## Results

### Statistical Analysis

#### Factor Analysis

Table 1 presents the KMO and Bartlett sphericity tests. The KMO measure assesses the suitability of the data for factor analysis and ranges from 0 to 1, with higher values indicating better suitability. Values of 0.631, 0.696, and 0.615 were generally considered acceptable for factor analysis. The Bartlett test of sphericity tests the null hypothesis that the intercorrelations among the variables are all equal to 0. It tests whether the observed correlation matrix is an identity matrix. If the test is significant, it indicates that the correlations among the variables are not all equal to zero and factor analysis may be appropriate. In this case, the tests were significant (*P*<.001), indicating that the factor analysis was appropriate for the data.

Tables 2-4 present the rotated component matrices for the haptic, visual, and postsession feedback variables, respectively. The rotated component matrix shows the factor loadings for each variable (haptic feedback 1, haptic feedback 2, etc) for each factor (component 1 and component 2). Factor loadings represent the degree to which each variable is associated with each factor. A factor loading of 0.7 or higher is considered a strong loading, while a factor loading between 0.4 and 0.7 is considered moderate. On the basis of these cutoffs, haptic feedback 1, 3, 5, and 6 had strong loadings on component 1, whereas haptic feedback 4 had a strong loading on segment 2. Haptic feedback 2 had a moderate loading on component 1, visual feedback 1-5 had strong loadings on component 1, whereas visual feedback 2 had a moderate loading on component 2 and postsession feedback 1, 2, 3, and 5 have strong loading on component 1, and postsession feedback 6 had moderate loading.

**Table 1:** Kaiser-Meyer-Olkin and Bartlett tests.

Test			Haptic feedback		Visual feedback		Postvisit feedback
Kaiser-Meyer-Olkin measure of sampling adequacy			0.631		0.696		0.615
**Bartlett test of sphericity**							
	Chi-square (*df*)	59.5 (15)		27.8 (15)		38.8 (15)	
	*P* value	<.001		.02		.001	

**Table 2:** Rotated component matrix (haptic feedback [HF]).

	Component 1	Component 2
HF1	0.935	—^a^
HF2	0.683	—
HF3	0.795	—
HF4	—	0.979
HF5	0.905	—
HF6	0.763	−0.459

^a^Not available.

**Table 3:** Rotated component matrix (visual feedback [VF]).

	Component 1	Component 2
VF1	0.805	—^a^
VF2	—	0.788
VF3	0.874	—
VF4	−0.401	0.749
VF5	0.776	—
VF6	0.707	—

^a^Not available.

**Table 4:** Rotated component matrix (postsession feedback [PF]).

	Component 1	Component 2
PF1	0.951	—^a^
PF2	0.728	—
PF3	0.702	—
PF4	—	−0.867
PF5	0.783	—
PF6	0.417	0.677

^a^Not available.

#### Reliability

Tables 5-7 provide statistics that can be used to evaluate the reliability of a scale or survey. Generally, a high corrected item-total correlation and a high Cronbach α are preferable. On the basis of Cronbach α values, haptic feedback 4, visual feedback 4, and postsession feedback 4 may be less reliable items, as they have a negative corrected item-total correlation, and deleting the items would result in a higher Cronbach α.

**Table 5:** Item-total statistics (haptic feedback [HF]).

	Scale mean if item deleted	Scale variance if item deleted	Corrected item-total correlation	Cronbach α if item deleted
HF1	43.11	312.693	0.867	.633
HF2	48.44	410.850	0.541	.734
HF3	44.39	350.840	0.681	.690
HF4	38.44	537.673	−0.198	.885
HF5	44.00	310.235	0.785	.651
HF6	44.67	333.176	0.601	.709

**Table 6:** Item-total statistics (visual feedback [VF]).

	Scale mean if item deleted	Scale variance if item deleted	Corrected item-total correlation	Cronbach α if item deleted
VF1	34.17	139.676	0.377	.431
VF2	38.33	172.353	0.321	.486
VF3	35.39	115.310	0.628	.275
VF4	27.17	215.559	−0.280	.762
VF5	35.61	150.958	0.576	.393
VF6	33.50	118.029	0.480	.357

**Table 7:** Item-total statistics (postsession feedback [PF]).

	Scale mean if item deleted	Scale variance if item deleted	Corrected item-total correlation	Cronbach α if item deleted
PF1	27.72	127.507	0.809	.391
PF2	29.44	154.614	0.516	.523
PF3	29.22	168.183	0.543	.542
PF4	19.50	197.324	−0.083	.768
PF5	27.33	145.765	0.575	.494
PF6	25.94	154.408	0.212	.657

### Test of Hypothesis

#### Prior Experience in Conducting Interviews Results in a Lower Cognitive Load

Table 8 shows the mean ratings for 3 different types of feedback (haptic, visual, and postsession) for the 2 groups: those with experience conducting interviews and those who did not. The haptic feedback rating for the group with interview experience was lower (4.84) than that for the group without interview experience (7.03). The visual feedback rating was almost similar for both groups (4.38 for those with experience, 4.22 for those without). However, the postvisit feedback rating was higher for the group with experience (3.41) compared with the group without experience (2.42). In the case of haptic feedback, the mean rating of the respondents with experience in conducting interviews was lower than that of those without experience, indicating that prior experience in conducting interviews results in a lower cognitive load.

Most participants with prior interview experience reported that although a learning curve was associated with haptic feedback, it allowed them to focus on their interpersonal skills, mainly speaking balance, pauses, and articulation. One participant claimed as follows:

The haptics were unintrusive. They were subtle enough, which helped me maintain my pace, yet distinctive enough when I needed to show improved speaking balance, enabling me to pause and ask questions.

Another participant stated as follows:

In most workplace settings, ongoing, targeted, and specific feedback are more powerful than post-session feedback as they allow you to make real-time improvements. Unlike post-session feedback, haptic feedback does not demand users to recollect past conversations or distract users with visual cues. Instead, it allows you to make real-time improvements to your interpersonal skills and helps you focus on maintaining body language and eye contact.

**Table 8:** Group statistics (haptic, visual, and postsession feedback).

Feedback and experience conducting interviews		Values, mean (SD; SE)
**Haptic feedback**		
	No (n=13)	7.0346 (3.99580; 1.10824)
	Yes (n=5)	4.8362 (3.31895; 1.48428)
**Visual feedback**		
	No (n=13)	4.2232 (2.17168; 0.60232)
	Yes (n=5)	4.3788 (2.97528; 1.33059)
**Postsession feedback**		
	No (n=13)	2.4179 (1.29804; 0.36001)
	Yes (n=5)	3.4054 (3.34773; 1.49715)

#### Correlation Between Prior Experience, Real-Time Feedback, and Performance

Tables 9 and 10 present the nonparametric test results to determine whether there is any correlation between prior experience in conducting interviews, real-time feedback, and performance.

**Table 9:** Nonparametric test results comparing the cognitive load between the respondents with experience conducting interviews and without experience conducting interviews.

Feedback and experience conducting interviews			Mean rank		Sum of ranks
**Haptic (n=18)**					
	0 (n=13)	10.38		135.00	
	1 (n=5)	7.20		36.00	
**Visual (n=18)**					
	0 (n=13)	9.62		125.00	
	1 (n=5)	9.20		46.00	
**Postsession (n=18)**					
	0 (n=13)	9.46		123.00	
	1 (n=5)	9.60		48.00	

**Table 10:** Wilcoxon inferences test results comparing the cognitive load between the respondents with experience conducting interviews and without experience conducting interviews.

	Haptic feedback	Visual feedback	Postsession feedback	Performance	Real-time feedback
Mann-Whitney *U*	21.000	31.000	32.000	6	6
Wilcoxon W	36.000	46.000	123.000	9	9
Z score	−1.134	−0.148	−0.049	−1.191	−1.189
*P* value (asymptotic significance; 2-tailed)	.26	.88	.96	.23	.23
*P* value (exact significance 2^a^; 1-tailed significance)	.29	.92	>.99	.30	.30

^a^Grouping variable: experience conducting interviews.

The findings from hypothesis A led to the assumption that participants with an interviewing experience who are subjected to real-time feedback (haptic and visual) will show improved performance compared with novice users because of the difference in the spare cognitive capacity that results from experience. A controlled experiment conducted by Zhou et al [14] previously tested this hypothesis with surgical residents. According to their findings, haptic feedback not only enhances performance but also counters the effect of cognitive loading, especially in the accuracy of task performance [14]. We attempted to test this hypothesis with our target group to determine whether the same findings were applicable to our study.

On the basis of these results, the haptic feedback group with no prior experience had the highest average rank, followed by the visual feedback and postvisit feedback groups. This suggests that, on average, participants with no experience conducting interviews had a higher ranking in haptic feedback compared with those without experience. However, when calculating the difference in means, the findings indicated that there was no significant difference between the means of haptic feedback, visual feedback, and real-time feedback (haptic+visual) between the 2 groups (*P*=.26, *P*=.88, and *P*=.23, respectively). In addition, there was no significant difference between the 2 groups in postsession feedback and performance (*P*=.96 and *P*=.23, respectively).

Although our findings contradict the hypothesis suggesting a correlation between prior experience in conducting interviews, real-time feedback, and performance, it is important to note that this finding could result from a small sample size (especially n=5 for respondents with prior experience).

#### The Performance of the Feedback Modality Largely Depends on the Cognitive Load Associated With the Feedback Tool

**Figure 5:**
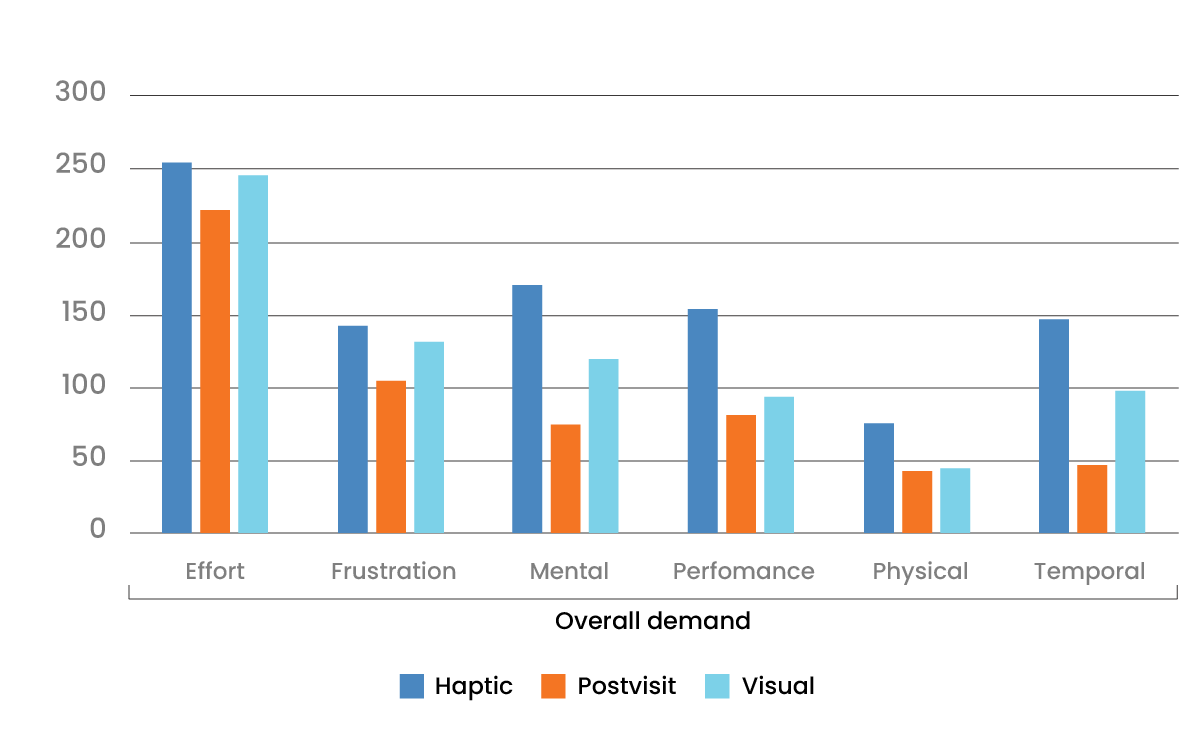
NASA Task Load Index findings comparing the cognitive load associated with each feedback.

On the basis of Figure 5, haptic feedback has the highest ratings for all demands, whereas postfeedback has the lowest ratings for these factors. The test presented below shows the differences in demand between the feedback modalities (Table 11).

The Kruskal-Wallis test is a nonparametric statistical test used to compare the medians of 2 or more groups. It is often used when the assumptions of the parametric 2 tailed *t* test or ANOVA are not met, such as when the data are not normally distributed or have a nonhomogeneous variance. The *P* value represents the probability of obtaining observed results if the null hypothesis is true. The null hypothesis is that there is no difference in the medians of the groups being compared, ie, haptic, visual, and postsession feedback modalities. A *P* value of less than .05 is typically considered statistically significant, meaning that the null hypothesis can be rejected and the results are likely not due to chance. On the basis of the above results, the mental and temporal demands between the 3 modalities are significantly different, with the highest demand being in the haptic modality group. Physical, effort, performance, and frustration demands were not significantly different between the groups. However, the postfeedback group had the lowest demand values.

The Wilcoxon signed-rank test, seen in Table 12, was used to compare the means of the postsession feedback and the haptic feedback, as well as the means of the postsession feedback and the visual feedback.

Table 12 shows the number and mean rank of negative ranks, positive ranks, and ties for each comparison. Negative ranks refer to cases in which the postsession feedback had a lower mean than the comparison (haptic or visual feedback). Positive ranks refer to cases where postsession feedback had a higher mean than the comparison group. Ties refer to cases in which the mean of the 2 groups was equal.

For the comparison between postsession feedback and haptic feedback, there were 15 negative ranks, 3 positive ranks, and 0 ties. This suggests that the mean of the postsession feedback group was lower than that of the haptic feedback group in most cases, but there were a few cases where the mean of the postsession feedback group was higher.

For the comparison between postsession feedback and visual feedback, there were 16 negative ranks, 2 positive ranks, and 0 ties. This suggests that the mean of the postsession feedback group was lower than that of the visual feedback group in most cases, but there were a few cases where the mean of the postsession feedback group was higher. Table 13 presents the test statistics.

For the comparison between postsession feedback and haptic feedback, the *Z* score was −3.245 and the *P* value was .001. This indicates that the null hypothesis can be rejected, and that there is a significant difference between the means of postsession feedback and haptic feedback, where postsession feedback had the lowest mean. For the comparison between postfeedback and visual feedback, the *Z* score was −2.940, and the *P* value was .003. This indicates that the null hypothesis can be rejected and that there is a significant difference between the means of postsession feedback and visual feedback, where visual feedback had the highest mean. For the comparison between postsession feedback and real-time feedback (haptic+visual), the difference was significant (*P*=.001), indicating that postfeedback modalities had a lower mean than real-time modalities. These findings indicate that feedback modalities with the lowest cognitive loads result in increased performance and efficacy.

**Table 11:** Kruskal-Wallis test results.

Feedback modality	Mean rank					
	Mental demand	Physical demand	Temporal demand	Effort	Performance	Frustration
Haptic (n=18)	35.47	33.03	36	30.39	33.86	29.14
Visual (n=18)	28.14	26.89	28.89	28.36	26.81	29.31
Postsession feedback (n=18)	18.89	22.58	17.61	23.75	21.83	24.06
*P* value	.006	.08	.002	.43	.07	.52

**Table 12:** Wilcoxon signed-ranks test results.

			Mean rank		Sum of ranks
**Postsession feedback: haptic feedback (n=18)**					
	Negative ranks (n=15)	10.67		160.00	
	Positive ranks (n=3)	3.67		11.00	
	Ties (n=0)	—^a^		—	
**Postsession feedback: visual feedback (n=18)**					
	Negative ranks (n=16)	9.56		153.00	
	Positive ranks (n=2)	9.00		18.00	
	Ties (n=0)	—		—	

^a^Not available.

**Table 13:** Test statistics based on positive ranks.

	Postsession feedback (haptic)	Postsession feedback (visual)	Postsession feedback (real time)
Z score	−3.245^a^	−2.940^a^	−3.419^a^
*P* value (asymptotic significance; 2-tailed)	.001	.003	.001

^a^Based on positive ranks.

## Discussion

### Overview

This study sought to investigate the efficacy of the 3 feedback tools while measuring their cognitive load in nonclinical participants in a nonclinical setting. The findings demonstrated that postsession feedback (received at the end of the interview session) was the feedback tool that caused the lowest cognitive load as opposed to real-time feedback modalities (received during the interviewing session). An individual’s performance largely coincided with the cognitive load associated with the feedback tool. Through our study, we discovered that cognitive workload moderates the relationship between the efficacy of feedback tools and their impact on performance (speaking balance and pace) such that their association becomes weaker or stronger depending on how high or low an individual’s cognitive load rating is while using the feedback tool. Consequently, the lower the cognitive load caused by the feedback tool, the better the performance and efficacy of the feedback tool, and vice versa.

The analysis of the poststudy survey also raised several important implications. The findings of the poststudy survey are discussed in the *Principal Findings* section.

### Principal Findings

#### Feedback Is Most Effective When It Is Improvement Focused

The results demonstrate that receiving feedback at the end of the session was more effective than receiving feedback during the session. In our study, real-time feedback concerning pace, articulation, and speaking balance was extensively provided through visual cues and haptics. In the postsession feedback, feedback was conveyed in the form of a written summary that included ratings, success criteria, and a performance summary.

Gamlem et al [15] state that users perceive feedback to be the most effective when it includes improvement-focused information that clarifies the next steps for learning. In our study, it was notable that most users found postsession feedback to be descriptive as it highlighted specific areas for improvement and explained why they received a particular rating or score. In contrast, real-time feedback tools provided evaluative feedback and failed to aid in long-term performance improvement. Users seeking performance improvement preferred feedback that helped them answer questions such as “What went wrong,” “What we learned today,” and “What could have been done better” [16]. These findings suggest that the innate quality of feedback makes the tool more effective than the timing of feedback (during or after the session). According to one participant, “Effective feedback helps promote personal and professional growth by offering continuous support, highlighting areas of improvement, and conveying correct standards of performance so that individuals can work toward improvement.”

#### Preference Toward the Feedback Tool Largely Depends on the Learning Style

The VARK (visual, aural, read or write, and kinesthetic) model developed by Fleming and Mills suggests that learning styles depend largely on the sensory modalities involved in understanding and processing information [17]. According to this model, visual learners process information best if they can see it. Auditory learners prefer to hear information; read-or-write learners prefer to see written words; and kinesthetic learners acquire knowledge through active participation.

According to the poststudy survey, the participant’s preference for the feedback tool coincided with their learning styles and impacted how they perceived and received the feedback given. This study found that 78% (14/18) of the study participants had multimodal learning style preferences and only 22% (4/18) had unimodal preferences. Among the multimodal learning styles, the most preferred mode was bimodal: 39% (7/18) suggested combining visual feedback with postsession feedback, 28% (5/18) desired a combination of haptic feedback with postsession feedback, and 11% (2/18) suggested a combination of visual feedback and haptic feedback.

The majority (14/18, 78%) of the users exhibited multimodal learning style preferences, indicating that users respond to feedback effectively as long as the feedback methods include a blend of activities that stimulate the VARK sensory modalities. Knowledge of individuals’ learning styles has implications for designing and developing practical feedback tools tailored to meet physicians’ learning preferences, as it directly impacts their performance.

One of the limitations of this study was its relatively small sample size. Therefore, these findings cannot be generalized to all health care staff and physicians. Further studies need to be conducted to examine the correlation between performance using feedback modalities and the learning styles of physicians. This would help us to further explore the possibility of combining 2 or more feedback modalities and testing their efficacy.

#### The Need for Real-Time Feedback Modalities to Offer Customization

Knowledge of personalizing real-time feedback modalities to improve interpersonal communication is largely underdeveloped. Understanding how different users perceive and respond to real-time feedback can help to develop effective feedback modalities. In the case of haptic feedback, vibrotactile stimulation creates only a rudimentary tactile output without a meaningful feedback loop. This configuration rarely accounts for the environmental noise. Our findings indicate that the impact of real-time feedback varies depending on the device and the setting. As stated by a user, “Haptic waveform effects can be easily perceivable, but sudden long vibrations can startle the user if played in a quiet environment such as a clinical setting or an interview setting.” When testing haptic feedback on mobile devices, there are fewer instances. Specific waveforms or rhythms can be misinterpreted as text or call notification. As a result, careful consideration must be taken to ensure that haptics are distinct. In addition, vibrations with increasing intensities or pulse waveforms can distract the user from their intended task, which may cause the user to turn off all haptics quickly. However, well-crafted haptics provides valuable sensory feedback, giving users richer engagement with their devices.

Our study findings indicate that 78% (14/18) of the users desired the ability to customize real-time feedback modalities. A total of 61% (11/18) of users expressed the desire to customize the sharpness and intensity of the haptics, whereas 28% (5/18) expressed a need to choose their preferred waveform and rhythm. According to the participants, different users have different tolerance levels for tactile feedback. Some waveforms convey a soft and calming experience, whereas others are either sharp or mechanical. The different haptic rhythms and intensities represent a wide range of emotions.

Notably, the preference for haptic rhythms, waveforms, and intensities also depends on the environment and setting. Users can explore various haptic and visual feedback experiences through customization to determine which option works best in a given environment or setting. As reported by the user, “The preference of a real-time feedback modality largely depends on the user’s environment. In stressful environments, such as clinical settings, I would like the ability to customize vibration patterns and intensities in a way that allows me to receive feedback in a calm and relaxed manner.” Similar findings were observed when visual feedback was tested. When asked about their preferred feedback tool, one participant responded, “I think my preference would change depending on my environment. Visual cues like transitioning or flashing colors may be effective in web-based environments. However, they may not have the same effect when applied to in-person clinical settings. In the case of web-based settings, visual cues can be unintrusive and beneficial but may be distracting during in-person conversations.”

The above feedback suggests the need to explore (1) whether customization of real-time feedback modalities helps improve its efficacy and (2) the impact of real-time feedback modalities in different environments (web-based and in-person) and its association with cognitive load. Additional features must be explored to optimize the effects and acceptability of feedback tools. For example, visual and tactile cues can be implemented to determine whether different types of feedback or a combination of feedback can counteract physicians’ burnout. More participants can be recruited to determine whether the preference for real-time feedback tools varies in web-based and in-person settings. Finally, real-time feedback tools must be customized to provide effective, evaluative, and descriptive feedback without negatively influencing the cognitive load associated with becoming accustomed to new feedback tools.

### Strengths and Limitations

By investigating multiple feedback tools, this study yields valuable insights into their comparative effectiveness and suitability for nonclinical scenarios. Conducting the study in a nonclinical setting allowed for better control over variables and reduced potential confounding factors related to medical conditions or clinical contexts.

Telemedicine, particularly via videoconferencing platforms, has gained prominence as a prevalent mode of communication between physicians and patients. Using Zoom for this study ensures that the findings are highly applicable to the current health care landscape, where telemedicine plays a significant role in facilitating remote consultation. Furthermore, using Zoom allows for an examination of cognitive load during telemedicine consultations, shedding light on the challenges and potential distractions encountered by physicians in web-based health care delivery. As a result, the study’s outcomes hold considerable relevance for telemedicine practices, providing valuable insights into optimizing physician-patient interactions and refining the implementation of feedback tools to enhance communication efficacy and alleviate cognitive load in remote medical encounters.

Nevertheless, it is imperative to acknowledge the inherent limitations of this study. The nonclinical setting used in this study may not accurately mimic real-world clinical scenarios, affecting the transferability of the findings to actual medical practices. Although the experimental setting serves as a valuable pilot or proof-of-concept study, it may not entirely determine the effectiveness of the feedback tools in clinical settings. The use of nonclinical participants limits the generalizability of the findings to clinical settings.

In addition, the use of Zoom to test feedback tools poses significant challenges that could influence study outcomes. Zoom is a videoconferencing platform that lacks the physical presence of participants. The absence of face-to-face interaction might compromise the authenticity and reliability of the feedback tool’s performance. Technical issues such as audio or video delays or glitches can lead to communication barriers. These barriers could negatively impact the effectiveness of real-time feedback, as participants may not fully comprehend the conveyed information because of interruptions or distortions. Second, Zoom might not effectively capture subtle nonverbal cues. These cues play a crucial role in effective communication, and their absence can hinder the evaluation of feedback tools, especially in terms of enhancing communication skills. Finally, Zoom sessions can be mentally taxing, especially in research contexts where participants are required to multitask between the platform and the feedback tools. The high cognitive load induced by the technology itself may interfere with participants’ focus and attention, potentially skewing the efficacy evaluation results.

To overcome these limitations, future research should address these concerns and expand the scope of this study. The inclusion of diverse participant groups and conducting research in clinical settings can provide more robust insights into the application of feedback tools in clinical settings. It is essential to explore other platforms or methods that better capture face-to-face interactions and nonverbal cues to enhance the authenticity of feedback tool evaluations. By acknowledging and working toward mitigating these limitations, future studies can contribute to a more comprehensive understanding of the effectiveness of feedback tools in clinical practice.

### Conclusions

This study highlights the potential of bimodal feedback tools to enhance physician-patient interactions, demonstrating the need for more extensive investigations in clinical environments. The integration of both real-time and postsession feedback presents a promising approach for enhancing physician-patient communication. Notably, postsession feedback not only improves performance but also mitigates the impact of cognitive loading. Our study demonstrated that postsession feedback contributes to the enhancement of verbal communication aspects, such as speaking balance, pace, and articulation. However, it is noteworthy that postsession feedback lacks specificity in addressing nonverbal competencies, including voice tone, body movement, facial expression, and eye contact, which can be better addressed through real-time feedback modalities [18-20]. To encourage empathic and patient-centered communication by health care professionals, future research is imperative to investigate the effectiveness of real-time and postsession feedback in both the verbal and nonverbal communication domains. We acknowledge the exploratory nature of this research while recognizing its contribution to identifying key factors that warrant further exploration in clinical scenarios. Subsequent studies in clinical settings will allow a comprehensive assessment of the efficacy and practical implementation of feedback tools in physician-patient interactions.

## Abbreviations

KMO: Kaiser-Meyer-Olkin
NASA TLX: NASA Task Load Index
PREM: patient-reported experience measure
VARK: visual, aural, read or write, and kinesthetic

## References

[1] (2017). The CAHPS ambulatory care improvement guide: practical strategies for improving patient experience. Agency for Healthcare Research and Quality.

[2] West CP, Dyrbye LN, Erwin PJ, Shanafelt TD (2016). Interventions to prevent and reduce physician burnout: a systematic review and meta-analysis. The Lancet.

[3] Young RA, Burge SK, Kumar KA, Wilson JM, Ortiz DF (2018). A time-motion study of primary care physicians' work in the electronic health record era. Fam Med.

[4] Marques-Pinto A, Moreira S, Costa-Lopes R, Zózimo N, Vala J (2021). Predictors of burnout among physicians: evidence from a national study in Portugal. Front Psychol.

[5] Harry E, Sinsky C, Dyrbye LN, Makowski MS, Trockel M, Tutty M, Carlasare LE, West CP, Shanafelt TD (2021). Physician task load and the risk of burnout among US physicians in a national survey. Jt Comm J Qual Patient Saf.

[6] Ashley L, Enid M (2021). Human-centered design reflections on providing feedback to primary care physicians. Proceedings of the Thematic Area, HCI 2021, Held as Part of the 23rd HCI International Conference on Human-Computer Interaction, Design and User Experience Case Studies.

[7] Shunmuga Sundaram C, Campbell R, Ju A, King MT, Rutherford C (2022). Patient and healthcare provider perceptions on using patient-reported experience measures (PREMs) in routine clinical care: a systematic review of qualitative studies. J Patient Rep Outcomes.

[8] Wolff AC, Dresselhuis A, Hejazi S, Dixon D, Gibson D, Howard AF, Liva S, Astle B, Reimer-Kirkham S, Noonan VK, Edwards L (2021). Healthcare provider characteristics that influence the implementation of individual-level patient-centered outcome measure (PROM) and patient-reported experience measure (PREM) data across practice settings: a protocol for a mixed methods systematic review with a narrative synthesis. Syst Rev.

[9] Farrington C, Burt J, Boiko O, Campbell J, Roland M (2017). Doctors' engagements with patient experience surveys in primary and secondary care: a qualitative study. Health Expect.

[10] Faucett HA, Lee ML, Carter S (2017). I should listen more: real-time sensing and feedback of non-verbal communication in video telehealth. Proc ACM Hum Comput Interact.

[11] NASA TLX: task load index. National Aeronautics and Space Administration.

[12] Pasquesi J, Gorlewicz JL (2021). Investigating multi-touch vibrations on mobile touchscreens. Proceedings of the 2021 IEEE World Haptics Conference.

[13] Xu M, Tachibana T, Suzuki N, Hoshino E, Terasawa Y, Miki N, Minagawa Y (2021). The effect of haptic stimulation simulating heartbeats on the regulation of physiological responses and prosocial behavior under stress: the influence of interoceptive accuracy. Biol Psychol.

[14] Zhou M, Jones DB, Schwaitzberg SD, Cao CG (2016). Role of haptic feedback and cognitive load in surgical skill acquisition. Proc Hum Factors Ergon Soc Annu Meet.

[15] Gamlem SM, Smith K (2013). Student perceptions of classroom feedback. Assess Educ: Princ Policy Pract.

[16] Brooks C, Carroll A, Gillies RM, Hattie J (2019). A matrix of Feedback for learning. Aust J Teach Educ.

[17] Fleming ND, Mills C (2008). VARK a guide to learning styles: VARK learn limited. VARK Learn.

[18] Patel RA, Hartzler A, Pratt WM, Back A, Czerwinski M, Roseway A (2013). Visual feedback on nonverbal communication: a design exploration with healthcare professionals. Proceedings of the 7th International Conference on Pervasive Computing Technologies for Healthcare.

[19] Hartzler AL, Patel RA, Czerwinski M, Pratt W, Roseway A, Chandrasekaran N, Back A (2014). Real-time feedback on nonverbal clinical communication. Theoretical framework and clinician acceptance of ambient visual design. Methods Inf Med.

[20] Stanton NA, Salmon PM, Walker GH, Baber C, Jenkins DP, Stanton NA (2005). Mental workload assessment methods. Human Factors Methods: A Practical Guide for Engineering and Design.

